# Oculo-orbital complications of odontogenic sinusitis

**DOI:** 10.22336/rjo.2023.30

**Published:** 2023

**Authors:** Mihai Alexandru Preda, Ovidiu Muşat, Caius Codruţ Sarafoleanu, Ioana Stella Popescu, Andreea Muşat, Ruxandra Pîrvulescu, Ramona Barac, Călin Petru Tătaru, Gabriela Cornelia Muşat

**Affiliations:** * “Carol Davila” University of Medicine and Pharmacy, Bucharest, Romania; ** “Dr. Carol Davila” Central Military Emergency Hospital, Bucharest, Romania; *** “Sfanta Maria” Clinical Hospital, Bucharest, Romania; **** Clinical Emergency Eye Hospital, Bucharest, Romania; ***** University Emergency Hospital, Bucharest, Romania

**Keywords:** odontogenic sinusitis, oculo-orbital complications, Chandler classification

## Abstract

**Introduction:** Odontogenic sinusitis is a well-known, but under-studied bacterial infection of the maxillary sinus that can extend to other sinuses, the orbit, or even the endocranium.

**Material and methods:** We performed an observational retrospective study on the patients with odontogenic sinusitis treated in our hospital over a five-year period. We included patients over 18 years old diagnosed with odontogenic sinusitis and ocular complications and we excluded patients with ocular complications nonrelated to dental-originated sinusitis or patients with odontogenic sinusitis without orbital-ocular complications.

**Results:** We examined the charts of 46 patients. From the total number of patients with oculo-orbital complications generated by odontogenic sinusitis, only 7 were women. The mean age was 33,7 with a standard deviation of 15,7 years. The oculo-orbital complications were assessed according to the Chandler classification. The most frequent orbital complication was preseptal cellulitis followed by orbital cellulitis. All the patients were treated with antibiotic covering both anaerobic and aerobic bacteria and 40 of the patients in our study received surgical treatment. The outcomes were favorable for all the patients in our study with clinical resolution.

**Conclusion:** The oculo-orbital complications of odontogenic sinusitis are severe because they can result in vision loss or other ocular sequelae. The bacteriological features of this sinusitis explain the special characteristics of this infection and can facilitate the extent of the infection to the orbit. Prompt intervention with antibiotics covering anaerobic and aerobic bacteria and surgery addressed to the affected sinus/ sinuses, the dental disease and the orbital pathology ensures a big success rate in the therapy of these complications.

## Introduction

Odontogenic sinusitis is a bacterial infection that originates in a dental pathology or following a dental procedure that involves the maxillary sinus and can extend to other sinuses [**1**]. The disease has been well recognized, but unfortunately not sufficiently studied [**2**]. The subject is not covered in international guidelines and position papers on rhinosinusitis [**3**,**4**]. The diagnosis of odontogenic sinusitis is sometimes difficult, requiring both otorhinolaryngological examination and dental evaluation. Some features of the disease might reveal the dental origin: the unilaterality of the disease and the microbiologic characteristics (polymorph flora with anaerobic bacteria) [**5**]. The disease’s special traits explain that odontogenic sinusitis has a high risk of being complicated by the oculo-orbital extension of the infection. The ophthalmologic complications of odontogenic sinusitis are severe, leading to vision loss or other unwanted ocular sequelae.

## Clinical study

The study aims to assess the characteristics of the oculo-orbital complications in case of sinusitis of dental origin.

We conducted a retrospective study examining the charts of patients admitted to the ENT department of the “Sf. Maria” Hospital in Bucharest during 5 years, between 2018-2022.

We included patients over 18 years old diagnosed with odontogenic sinusitis and ocular complications treated in our department, we excluded patients with ocular complications nonrelated to dental-originated rhinosinusitis or patients with odontogenic sinusitis without orbito-ocular complications. 

We evaluated the patients from the point of view of the history, symptoms, clinical and endoscopic examination, ophthalmologic involvement, bacteriologic cultures, CT imaging, dental involvement, medical and surgical treatment, and outcomes.

The oculo-orbital complications were evaluated according to the Chandler classification [**6**]. This classification includes five stages:

1. preseptal cellulitis: inflammation and edema anterior to the orbital septum;

2. orbital cellulitis: inflammation and edema posterior to the orbital septum;

3. subperiosteal abscess between the lamina papyracea and the orbital periosteum;

4. orbital abscess;

5. cavernous sinus thrombosis.

The dental pathology that generated the orbito-ocular complication was evaluated by a dentist. Two major dental pathologies were linked to sinusitis complicated with the orbital disease: 

• periodontitis;

• dentigerous cyst.

We also included the sinusitis produced by dental surgical interventions:

• dental extractions;

• foreign body (including dental implant); 

• root canal therapy;

• oro antral fistulas.

During the five-year period, 2029 patients were admitted to the ENT department with the diagnosis of sinusitis. 517 of those patients were diagnosed with odontogenic sinusitis (25,48%).

Of the 517 patients with odontogenic sinusitis, 46 patients were diagnosed with oculo-orbital complications (8,89%). All the complications were ipsilateral with the dental pathology.

The diagnosis was based on the ENT clinical and endoscopic examination, the CT scan of the sinuses, and the dental examination showing the originating tooth pathology. An ophthalmologist performed the ocular examination to assess the orbito-ocular complications.

The symptoms the patients reported were facial pain, nasal discharge, nasal obstruction, cacosmia, dental pain, blurred vision, and diplopia. Dental pain was reported in 38% of the patients.

The following medical problems were observed at the physical examination of the patients: exophthalmia, eyelid edema, ocular motility troubles, chemosis, congestion of the conjunctiva, and a decrease in visual acuity (5%). At the nasal examination, we observed nasal mucosa congestion and purulent secretions in the middle meatus in some patients. An interesting finding was the fact that a high number of the patients with dental sinusitis (26, 56,5%) did not have purulent discharge in the middle meatus. 

From the total of 46 patients with oculo-orbital complications generated by odontogenic sinusitis, only 7 were women. As for the age distribution, we noticed that most of the patients were young. The mean age was 33,7 with a standard deviation of 15,7 years.

The dental and radiologic examination of the patients in the study showed periapical abscess in 86% of cases and periodontal abscess in 13% of cases and a dentigerous cyst in one case. As for the tooth that generated the sinus pathology, usually, it was a pathology generated mainly by the molars. We found that molars were involved in 86,95% of cases followed by premolars in 10,88% of cases, and canines in 2,17% of cases. 

The CT findings for the patients with odontogenic rhinosinusitis were relevant for the extent of the disease showing different degrees of involvement from mild thickening of the mucosal layer to the opacification of multiple sinuses, even pansinusitis. 

**Fig. 1:**
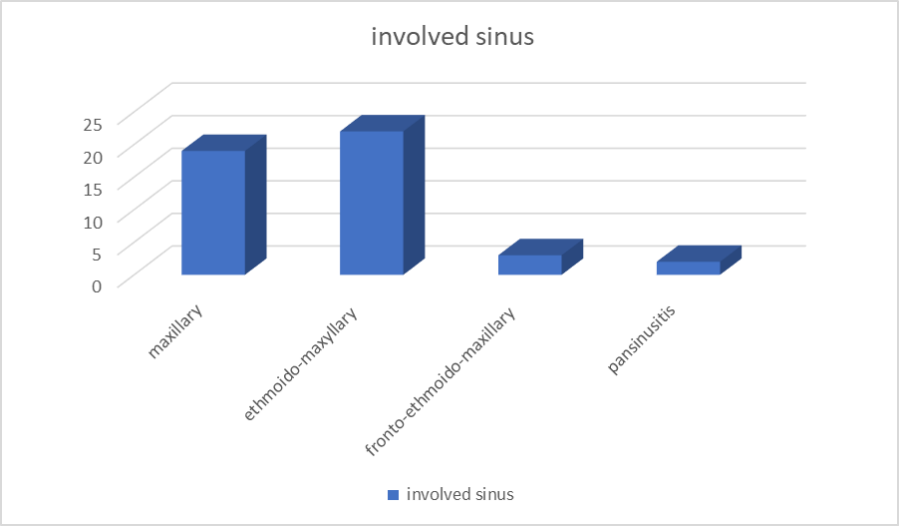
Distribution according to the sinus involvement for the patients with odontogenic sinusitis and oculo-orbital complications

Considering the involved sinuses, the following distribution was found: maxillary sinusitis 41,3%, ethmoid-maxillary sinusitis 47,8%, frontal-ethmoid-maxillary sinusitis 6,5%, pansinusitis 4,3% (see **Fig. 1**).

**Fig. 2:**
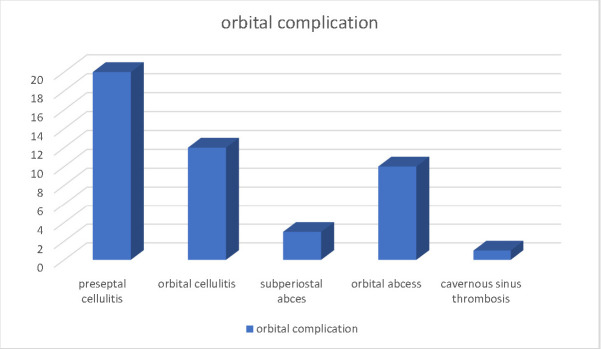
Distribution of the orbital complications for the study group

Considering the Chandler classification, the distribution of the orbital complications was the following: preseptal cellulitis 43,4%, orbital cellulitis 26%, subperiosteal abscess 6,5%, orbital abscess 21,7%, cavernous sinus thrombosis 2,1% (see **Fig. 2**).

An interesting finding was the fact that although we collected samples for bacteriological examination from sinus and orbital secretions at admission and during surgery, the laboratory did not find pathogens in 40 cases. In the rest of the cases, pseudomonas aeruginosa, staphylococcus aureus, and streptococcus species were identified. 

The treatment of the orbital complications consisted in antibiotherapy associated with steroid anti-inflammatory and surgical treatment addressed to the sinusitis and the orbital pathology. Of the 46 patients with oculo-orbital complications in the study, 40 patients underwent surgery. 35 of these patients were operated using an endoscopic approach for the involved sinus and the affected orbit (drainage of the orbital abscess or subperiosteal abscess). Another 5 patients were operated using a combined approach - endoscopic and external. An orbital decompression was performed for 20 patients. All the patients were treated with extensive antibiotherapy covering aerobic and anaerobic bacteria. The antibiotherapy was maintained until the clinical resolution and two weeks afterwards.

The dental pathology was treated by dental surgeons during their stay in the hospital, in a second step after the ENT surgery. 

We monitored the patients postoperatively for rhinologic, ophthalmologic, and dental signs. The surveillance was usually conducted for one year. 

The patients in our study had positive results in maintaining their visual acuity after the treatment.

## Discussion

Data showed that oculo-orbital complications represent approximately 80% of rhinosinusitis complications. It is considered that 60-70% of the infective-inflammatory pathology of the orbit is consecutive to a sinus infection [**7**]. Orbito-ocular complications of odontogenic sinusitis tend to be more severe than those of rhinogenic sinusitis, possibly due to the anaerobic bacteria involved. 

The oculo-orbital complications of rhinosinusitis are generally determined by ethmoidal rhinosinusitis [**3**], but odontogenic maxillary sinusitis tends to extend to the orbit more frequently than generally expected. In our study, the oculo-orbital complications did not have any correlation with the extent of the disease. This might be explained by the fact that the dental infection might extend to the orbit through other pathways except for the sinus. It is possible that there might be a hematogenous way through the veins in the maxillary alveolar marrow spaces or maybe submucosal through oral and facial veins [**8**]. Most of the patients with orbito-ocular complications we considered in our study were patients that had only maxillary sinusitis 41,3%. This might be an indicator of the fact that the extension of the infection is mostly produced through a hematogenous way or maybe pre-existent anatomic dehiscences.

This paper presented our experience in the management of ocular complications of odontogenic sinusitis. This pathology is not covered so well by the guidelines and international position papers although it is quite frequent. In our study, we established that 25.48% of the total number of rhinosinusitis treated in our department were odontogenic sinusitis. This proportion was within the limits of the literature reports for odontogenic sinusitis. In their study, Melen et al. found a dental cause for rhinosinusitis in 40,6% of cases [**9**]. In another study from 2020, the proportion was less important: out of 411 patients with rhinosinusitis, 74 were diagnosed with odontogenic rhinosinusitis, and 30 with oroantral fistula [**10**]. In their study, Maillet et al. established the fact that more than 50% of the changes in the maxillary sinuses seemed to be associated with periapical pathology [**11**].

Of the total number of patients with odontogenic sinusitis, 46 were admitted presenting an oculo-orbital complication of the disease. This represented 8.89% of the patients with odontogenic sinusitis. We did not find any published data to compare the frequency we found with other studies. 100% of the complications were ipsilateral, generating dental pathology. If we consider the tooth originating to the sinus pathology, the results of our study were similar to those found in other studies on the same subject. There are studies showing that the first and second molars are 11 times more likely to be implicated than the premolars [**11**].

Regarding the bacteria implicated in the physiopathology of odontogenic sinusitis, literature data underline that anaerobes are prevalent, particularly Porphyromonas, Peptostreptococcus, Prevotella, and Fusobacterium, but we can also find Gram-positive and Gram-negative aerobes in the cultures [**12**]. It is considered that odontogenic rhinosinusitis is a polymicrobial infection gathering bacteria from the oral cavity and from the respiratory tract with a predominance of anaerobes [**13**]. There are significant differences between the bacteriological features of rhinogenic and odontogenic sinusitis [**14**]. Although we cultured samples from different anatomic locations (orbit, sinus, nasal cavity), the cultures were negative in most cases. We interpreted this by the inability of the laboratory to identify anaerobes. 

Similar to the data found in the sinusitis literature, the oculo-orbital complications of sinusitis occur mostly in men and young adults [**15**,**16**]. Unlike the case of rhinogenic sinusitis, oculo-orbital complications of odontogenic sinusitis occur more frequently in adults [**17**]. In our study, we did not include patients under 18 years old, so we could not assess the differences between different ages. The fact that young age is more affected by this pathology is very interesting and needs to be further studied.

From a total of 46 cases of complicated sinusitis, only 7 were women. This is consistent with the fact that in literature, the proportion of male patients with oculo-orbital complications linked to odontogenic rhinosinusitis is more important than the female one.

It seems that the oculo-orbital complications of odontogenic sinusitis are more prone to result in vision loss than rhinogenic sinusitis. The vision loss from non-odontogenic rhinosinusitis was situated between 0 and 12% of the patients [**18**]. In contrast, odontogenic rhinosinusitis tends to result in vision loss more than rhinogenic sinusitis [**8**]. In our study, none of the patients lost their visual acuity. This might be a result of the fact that in all the patients the intervention was prompt with antibiotics with anaerobe coverage and appropriate surgical therapy.

## Conclusions

Oculo-orbital complications of odontogenic sinusitis can be severe, resulting in vision loss and unwanted ocular sequelae. The bacteriological features of this sinusitis explain the special characteristics of this infection and possibly the reason the infection can extend the orbit. Whenever the pathology is unilateral, a dental cause should be suspected and investigated. We noticed that from the total number of patients in our study, a major part were young male adults. We consider this to be a direction of research for further studies. Prompt intervention with antibiotics covering anaerobic and aerobic infections and surgery addressed to the affected sinus/ sinuses and the orbital pathology ensure a favorable outcome in the therapy of these complications.

**Conflict of Interest statement**

The authors state no conflict of interest.

**Informed Consent and Human and Animal Rights statement**

Informed consent has been obtained from all individuals included in this study.

**Authorization for the use of human subjects**

Ethical approval: The research related to human use complies with all the relevant national regulations, institutional policies, is in accordance with the tenets of the Helsinki Declaration, and has been approved by the review board of “Dr. Carol Davila” Central Military Emergency Hospital, Bucharest, Romania.

**Acknowledgements**

None.

**Sources of Funding**

None.

**Disclosures**

None.

**Competing interests**

None.
